# A phase I/II trial of beta-(1,3)/(1,6) D-glucan in the treatment of patients with advanced malignancies receiving chemotherapy

**DOI:** 10.1186/1756-9966-27-40

**Published:** 2008-09-19

**Authors:** Alan B Weitberg

**Affiliations:** 1 Department of Medicine, Roger Williams Medical Center, Providence, Rhode Island and Boston University School of Medicine, Boston, Massachusetts, USA

## Abstract

β-(1,3)/(1,6) D-glucan, a component of the fungal cell wall, has been shown to stimulate the immune system, enhance hematopoiesis, amplify killing of opsonized tumor cells and increase neutrophil chemotaxis and adhesion. In view of these attributes, the β-glucans should be studied for both their therapeutic efficacy in patients with cancer as well as an adjunctive therapy in patients receiving chemotherapy as a maneuver to limit suppression of hematopoiesis.

In this study, twenty patients with advanced malignancies receiving chemotherapy were given a β-(1,3)/(1,6) D-glucan preparation (MacroForce plus IP6, ImmuDyne, Inc.) and monitored for tolerability and effect on hematopoiesis. Our results lead us to conclude that β-glucan is well-tolerated in cancer patients receiving chemotherapy, may have a beneficial effect on hematopoiesis in these patients and should be studied further, especially in patients with chronic lymphocytic leukemia and lymphoma.

## Background

β-(1,3)/(1,6) D-glucan is a long chain polymer of glucose from the fungal cell wall which has been shown to have a number of immunomodulatory properties as well as effects on hematopoiesis and as a radiation protectant.

It has been well-demonstrated that the β-glucans increase neutrophil chemotaxis and adhesion, synergize with myeloid growth factors to enhance hematopoiesis and mobilize peripheral blood progenitor cells *in vivo*, directly stimulate committed myeloid progenitor cells and improve survival and hematopoietic regeneration in irradiated mice [1-7].

Furthermore, the β-glucans have been shown to amplify the phagocytic killing of opsonized tumor cells and combine with monoclonal antibodies to increase their tumoricidal activity [8].

Based on these properties, this study was designed to test the safety of an adjunctive treatment with β-(1,3)/(1,6) D-glucan in patients with advanced malignancies receiving chemotherapy. In addition, because the β-glucans have been shown to improve hematopoiesis in animals and because chemotherapy generally induces cytopenias in humans, we also sought to determine if the β-glucan administered in this study exerted an effect on blood counts in patients with advanced malignancies receiving chemotherapy compared with pretreatment blood counts in patients receiving chemotherapy alone.

## Methods

Twenty patients with advanced malignancies receiving chemotherapy received the β-(1,3)/(1,6) D-glucan preparation, MacroForce plus IP6 (ImmuDyne, Mount Kisco, NY), one tablet twice a day by mouth after granting informed consent. This study was approved by the ethics committee of the University Medical Group. All patients had received at least one course of chemotherapy prior to entering the study, were between 38 and 84 years old and had a performance status of 0 to 2, according to the criteria of the World Health Organization.

Patients were monitored every two weeks for side effects and complete blood counts were obtained monthly for six months. Specifically, patients were asked to report any new symptoms not experienced prior to initiation of the study and to note if any prior chemotherapy-related symptoms (e.g. nausea and vomiting) had changed after the intiation of the study. If grade 3 or 4 hematologic toxicity developed, defined as an absolute neutrophil count of less than 1000 uL or a platelet count of less than 50,000 uL, therapy would be withheld until the toxicity resolved. After resolution, dosing would be reduced to one tablet by mouth per day. Mean changes in blood counts during the study were compared to the mean blood counts observed prior to the initiation of the study.

The primary endpoint was to determine the tolerability of the treatment and its effect on serial blood counts. Written informed consent was obtained from each patient for publication of this study.

## Results

Table 1 demonstrates the characteristics of the patients enrolled in this study. Table 2 notes the types of malignancies experienced by these patients and the treatments they were receiving.

**Table 1:** Patient Characteristics

Characteristic	Study Patients (N = 20)
Median age – yrs (range)	65 (38–84)
	
Sex (no.)	
Male	10
Female	10
	
WHO Performance Score	
0	14
1	5
2	1

**Table 2:** Patients' Diagnoses and Treatments

Patient #	Diagnosis	Treatment
1	non-small cell lung cancer	carboplatin/taxol
2	pancreatic carcinoma	VP-16/gemzar
3	breast carcinoma	carboplatin/taxotere
4	breast carcinoma	adriamycin/taxotere
5	non-small cell lung cancer	carboplatin/taxol
6	non-small cell lung cancer	carboplatin/taxol
7	small cell lung cancer	VP-16/cisplatin
8	colon cancer	5 FU/leukovorin/oxaliplatin
9	breast cancer	adriamycin/taxotere
10	breast cancer	carboplatin/taxol
11	follicular lymphoma	cytoxan/vincristine/prednisone
12	renal cell carcinoma	cytoxan/nexavar
13	chondrosarcoma	cytoxan/adriamycin/ifosphamide
14	colon cancer	5 FU/leukovorin/oxaliplatin
15	breast cancer	adriamycin/taxotere
16	follicular lymphoma	cytoxan/vincristine/prednisone
17	chronic lymphocytic leukemia	alkeran/prednisone
18	chronic lymphocytic leukemia	alkeran/prednisone
19	pancreatic carcinoma	doxorubicin/gemzar
20	myelodysplastic syndrome	hydroxyurea

None of the twenty patients reported any new symptoms while taking the β-glucan. Sixty per cent of the patients reported a sense of well-being while taking the β-glucan and asked to remain on the treatment even after the completion of the study. Forty per cent of the patients who experienced fatigue during their chemotherapy treatments prior to entering the study reported feeling less fatigued while taking the β-glucan. In addition, one patient with lymphoma and significant cervical adenopathy who delayed his standard chemotherapy for 4 weeks during the study and only took the β-glucan, noted a marked reduction in the size of the nodes while taking the β-glucan alone.

Table 3 shows the comparison of the mean blood counts prior to entering the study with the mean blood counts during the study. As can be seen, in general, there was a trend to improved blood counts during the study as compared with the pre-study period. There appeared to be improvement especially of the white blood count during the study period on this dose of β-glucan. Improvements were also noted in the levels of hemoglobin and the platelet counts.

**Table 3:** Change in Mean Complete Blood Count Values

Patient #	White blood count (ul)	Hemoglobin (gm/dl)	Platelet count (ul)
1	+ 100	+.03	+26,000
2	+1100	+.30	+23,000
3	+1700	+1.0	+ 8,000
4	+1000	No change	-23,000
5	No change	No change	No change
6	+1800	-0.2	+15,000
7	No change	No change	No change
8	+ 600	+.60	No change
9	No change	No change	-4,000
10	+ 300	+.30	No change
11	+ 700	+.40	-21,000
12	No change	No change	No change
13	+ 300	No change	+33,000
14	No change	No change	No change
15	No change	No change	No change
16	+ 600	+.70	+ 3,000
17	+ 200	+.20	No change
18	+100	No change	No change
19	No change	No change	No change
20	+600	+.05	+ 5,500

## Discussion

The β-glucans are long-chain polymers of glucose in β-(1,3)(1,6) linkages which comprise the fungal cell wall. These glucans have been studied for their ability stimulate hematopoiesis, protect against radiation injury and amplify killing of opsonized tumor cells.

It has been shown that these glucans can increase neutrophil chemotaxis and adhesion in several *in vivo *and *in vitro *systems [1-3]. In addition, betafectin PGG-glucan was able to synergize with myeloid growth factors *in vivo *to enhance hematopoietic recovery and mobilize peripheral blood progenitor cells [4]. Treatment with glucans in mice has been shown to increase survival and improve hematopoietic regeneration after irradiation and that the glucans act directly on commited myeloid progenitors to improve hematopoiesis [5-7].

The effects of glucans on tumor cell killing has included the observation that yeast β-glucan amplifies phagocytic killing of iC3b-opsonized tumor cells, combine with monoclonal antibodies to increase tumor cell death and can increase macrophage cytotoxicity to tumor cells by increasing nitrous oxide production [8-12].

Interestingly, toxicological assessment of a particulate yeast (1,3)-β-D-glucan in rats revealed no adverse or toxic effects on the animals [13,14].

The specific aim of this study was to determine the safety and side effect profile of β-(1,3)/(1,6) D-glucan in patients with advanced malignancies receiving chemotherapy and the effect of the glucan on hemtopoiesis in these patients. Furthermore, because the β-glucans also have immunomodulatory effects as noted above, we monitored patients for any therapeutic effect of the glucan even though this was an uncontrolled trial and this was not a specific aim of the treatment paradigm.

This trial demonstrates that β-(1,3)/(1,6) D-glucan is extremely well-tolerated in patients with advanced malignancies receiving chemotherapy. No adverse effects or toxicities were reported by any of the patients when compared to their symptom profile before entering the study. On the contrary, a significant number of patients reported a sense of well-being while taking the glucan, an effect which should be studied further in a larger, controlled trial.

There clearly was some amelioration of the blood counts in patients taking the glucan as compared to the pretreatment mean counts. This effect supports the data from animal studies which demonstrates that the β-glucans improve hematopoiesis through a variety of mechanisms. This effect should also be studied in a larger, controlled trial because if confirmed, the β-glucans could become an important adjunctive treatment in patients receiving chemotherapy to limit the cytopenias observed with these therapies.

Lastly, because of the immunomodulatory properties of the glucans, these agents may have direct tumoricidal properties which deserve further investigation in a larger population in a controlled setting. Our anecdotal experience in one patient with lymphoma and enlarged cervical nodes who noted marked improvement on the β-glucan alone is extremely interesting at the very least.

We conclude that β-(1,3)/(1,6) D-glucan can be safely administered to patients with advanced malignancies receiving chemotherapy and that this adjunctive therapy may have beneficial effects on the blood counts in these patients. Further studies are warranted in a larger patient population to confirm this latter finding and to determine if the β-glucans have tumoricidal effects in humans.

## Competing Interests

The author declares that he has no competing interests.
